# *GBA* variants in REM sleep behavior disorder: A multicenter study

**DOI:** 10.1212/WNL.0000000000010042

**Published:** 2020-08-25

**Authors:** Lynne Krohn, Jennifer A. Ruskey, Uladzislau Rudakou, Etienne Leveille, Farnaz Asayesh, Michele T.M. Hu, Isabelle Arnulf, Yves Dauvilliers, Birgit Högl, Ambra Stefani, Christelle Charley Monaca, Beatriz Abril, Giuseppe Plazzi, Elena Antelmi, Luigi Ferini-Strambi, Anna Heidbreder, Bradley F. Boeve, Alberto J. Espay, Valérie Cochen De Cock, Brit Mollenhauer, Friederike Sixel-Döring, Claudia Trenkwalder, Karel Sonka, David Kemlink, Michela Figorilli, Monica Puligheddu, Femke Dijkstra, Mineke Viaene, Wolfgang Oertel, Marco Toffoli, Gian Luigi Gigli, Mariarosaria Valente, Jean-François Gagnon, Alex Desautels, Jacques Y. Montplaisir, Ronald B. Postuma, Guy A. Rouleau, Ziv Gan-Or

**Affiliations:** From the Department of Human Genetics (L.K., U.R., G.A.R., Z.G.-O.), Montreal Neurological Institute (L.K., J.A.R., U.R., E.L., F.A., R.B.P., G.A.R., Z.G.-O.), and Department of Neurology and Neurosurgery (J.A.R., F.A., R.B.P., G.A.R., Z.G.-O.), McGill University, Montréal; Oxford Parkinson's Disease Centre (M.T.M.H.) and Nuffield Department of Clinical Neurosciences (M.T.M.H.), University of Oxford, UK; Sleep Disorders Unit (I.A.), Pitié Salpêtrière Hospital, Centre de Recherche de l’Institut du Cerveau et de la Moelle Epinière and Sorbonne Universities, Paris; National Reference Center for Narcolepsy, Sleep Unit (Y.D.), Department of Neurology, Gui-de-Chauliac Hospital, CHU Montpellier, University of Montpellier, Inserm U1061, France; Sleep Disorders Unit, Department of Neurology (B.H., A.S.), Medical University of Innsbruck, Austria; Department of Clinical Neurophysiology and Sleep Center (C.C.M.), University Lille North of France, CHU Lille; Sleep Disorder Unit (B.A.), Carémeau Hospital, University Hospital of Nîmes, France; Department of Biomedical and Neuromotor Sciences (DIBINEM) (G.P., E.A.), Alma Mater Studiorum, University of Bologna; IRCCS (G.P., E.A.), Istituto delle Scienze Neurologiche, Bologna; Department of Neurological Sciences (L.F.-S.), Università Vita-Salute San Raffaele, Milan, Italy; Department of Neurology with Institute of Translational Neurology (A.H.), University of Muenster, Germany; Department of Neurology (B.F.B.), Mayo Clinic, Rochester, MN; UC Gardner Neuroscience Institute and Gardner Family Center for Parkinson's Disease and Movement Disorders (A.J.E.), Cincinnati, OH; Sleep and Neurology Unit (V.C.D.C.), Beau Soleil Clinic, Montpellier; EuroMov (V.C.D.C.), University of Montpellier, France; Paracelsus-Elena-Klinik (B.M., F.S.-D., C.T.), Kassel; Department of Neurology (B.M., C.T.), University Medical Centre Goettingen; Department of Neurology (F.S.-D., W.O.), Philipps University, Marburg, Germany; Department of Neurology and Centre of Clinical Neuroscience (K.S., D.K.), Charles University, First Faculty of Medicine and General University Hospital, Prague, Czech Republic; Department of Medical Sciences and Public Health, Sleep Disorder Research Center (M.F., M.P.), University of Cagliari, Italy; Laboratory for Sleep Disorders (F.D., M.V.) and Department of Neurology (F.D., M.V.), St. Dimpna Regional Hospital, Geel, Belgium; Department of Medicine (DAME) (M.T., M.V.), University of Udine, Italy; Department of Clinical and Movement Neurosciences (M.T.), UCL Queen Square Institute of Neurology, London, UK; Clinical Neurology Unit (G.L.G., M.V.), Department of Neurosciences, University Hospital of Udine; DMIF (G.L.G.), University of Udine, Italy; Centre d’Études Avancées en Médecine du Sommeil (J.-F.G., A.D., J.Y.M., R.B.P.), Hôpital du Sacré-Cœur de Montréal; and Departments of Psychology (J.-F.G.), Neurosciences (A.D.), and Psychiatry (J.Y.M.), Université du Québec à Montréal, Canada.

## Abstract

**Objective:**

To study the role of *GBA* variants in the risk for isolated REM sleep behavior disorder (iRBD) and conversion to overt neurodegeneration.

**Methods:**

A total of 4,147 individuals were included: 1,061 patients with iRBD and 3,086 controls. *GBA* was fully sequenced using molecular inversion probes and Sanger sequencing. We analyzed the effects of *GBA* variants on the risk of iRBD, age at onset (AAO), and conversion rates.

**Results:**

*GBA* variants were found in 9.5% of patients with iRBD compared to 4.1% of controls (odds ratio, 2.45; 95% confidence interval [CI], 1.87–3.22; *p* = 1 × 10^−10^). The estimated OR for mild p.N370S variant carriers was 3.69 (95% CI, 1.90–7.14; *p* = 3.5 × 10^−5^), while for severe variant carriers it was 17.55 (95% CI, 2.11–145.9; *p* = 0.0015). Carriers of severe *GBA* variants had an average AAO of 52.8 years, 7–8 years earlier than those with mild variants or noncarriers (*p* = 0.029). Of the *GBA* variant carriers with available data, 52.5% had converted, compared to 35.6% of noncarriers (*p* = 0.011), with a trend for faster conversion among severe *GBA* variant carriers. However, the results on AAO and conversion were based on small numbers and should be interpreted with caution.

**Conclusions:**

*GBA* variants robustly and differentially increase the risk of iRBD. The rate of conversion to neurodegeneration is also increased and may be faster among severe *GBA* variant carriers, although confirmation will be required in larger samples. Screening for RBD in healthy carriers of *GBA* variants should be studied as a potential way to identify *GBA* variant carriers who will develop a synucleinopathy in the future.

Isolated REM sleep behavior disorder (iRBD) can be considered a prodromal synucleinopathy, because >80% of patients with iRBD will eventually convert to an overt neurodegenerative syndrome associated with α-synuclein accumulation—Parkinson disease (PD), dementia with Lewy bodies (DLB), or multiple system atrophy (MSA)^1^—with a conversion rate of about 6% a year.^2^ For unknown reasons, while some patients with iRBD convert rapidly, others can remain free from parkinsonism or dementia for decades.^3,4^

Variants in the gene encoding for the lysosomal enzyme glucocerebrosidase, *GBA*, are strong and relatively common risk factors for PD^5,6^ and DLB,^7^ yet their role in MSA is unclear.^8–10^ Patients with PD who carry *GBA* variants, as a group, tend to have higher rates of nonmotor symptoms, including REM sleep behavior disorder (RBD), cognitive impairment, hyposmia, and autonomic dysfunction.^11^ *GBA* variants can be classified as severe or mild based on the type of Gaucher disease (GD) associated with the variant.^12^ Accordingly, patients with severe *GBA* variants have a higher risk for PD, an earlier average age at onset (AAO),^5^ and faster cognitive decline^13,14^ compared to patients with PD with mild or no *GBA* variants.

Few studies with small sample size have examined the role of *GBA* in iRBD, including studies of 69,^15^ 171,^16^ and 265 patients with iRBD,^15^ all supporting an association between *GBA* variants and iRBD but with different risk estimates. It has been shown that in PD cohorts with available data on probable RBD (pRBD), *GBA* variants are more frequent in the group with pRBD.^15^ However, there are no accurate estimates of the risk of iRBD among *GBA* variant carriers and there have been no studies separately analyzing severe and mild *GBA* variants. It is not clear whether *GBA* variants affect the rate of conversion from iRBD to overt synucleinopathies, as only 2 small sample size studies with contradicting results examined this hypothesis. In one, there was no association with the rate of conversion in 8 *GBA* variant carriers with iRBD^16^; in the other, a faster conversion was shown for 13 *GBA* variant carriers with iRBD compared to noncarriers.^17^

In this study, we analyzed *GBA* variants in a large, multicenter study including 1,061 patients with iRBD, more than double the sample than all previous studies combined, and 3,086 controls, all of European origin. We further examined the effects of severe vs mild *GBA* variants on risk of iRBD, reported AAO of iRBD, and the potential effects on conversion from iRBD to an overt neurodegenerative disease.

## Methods

### Population

The patient population included 1,061 individuals diagnosed with iRBD with video-polysomnography according to the International Classification of Sleep Disorders, version 2 or 3 criteria.^18^ The recruiting centers and the number of patients from each center are detailed in table 1. Additional data were available for subsets of samples, including reported AAO of RBD (n = 594), age at diagnosis of iRBD (n = 599), eventual phenoconversion to an overt neurodegenerative disease (data available for n = 584, converted n = 218), and rate of phenoconversion (n = 217). The average follow-up period for *GBA* carriers was 4.8 years and for noncarriers it was 4.1 years (*p* = 0.22).

**Table 1:**
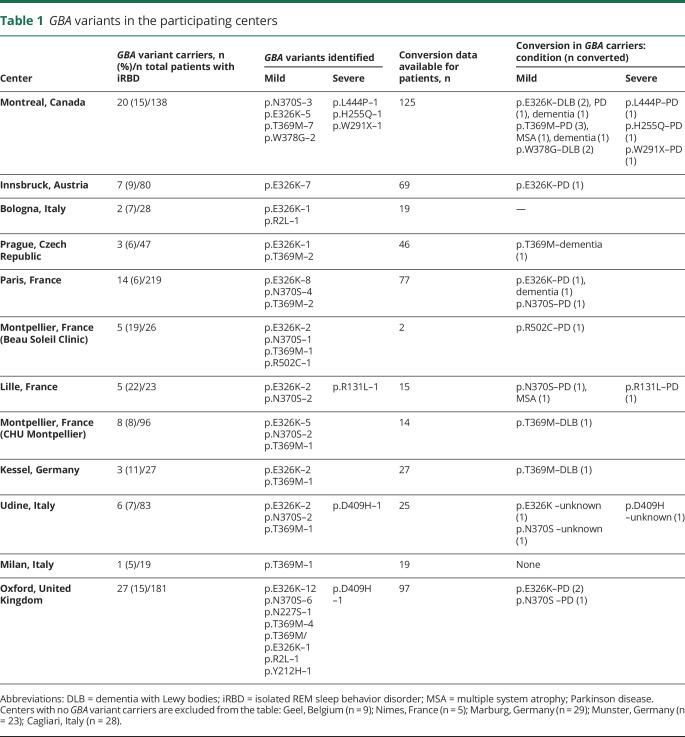
*GBA* variants in the participating centers

The data on these variables were collected in 2018. The control population included a total of 3,086 individuals, comprised of 1,317 in-house controls of European origin (confirmed by principal component analysis using available genome-wide association study [GWAS] data compared to data from HapMap v.3 and hg19/GRCh37) and an additional 1,769 previously published European controls in which *GBA* was fully sequenced and all the variants were reported (table e-1 details these controls and the reported *GBA* variants in each of the articles; github.com/gan-orlab/GBA_RBD/). The in-house controls had a mean age of 46.5 ± 15.0 years and included 46.6% men, compared to 60.5 ± 9.9 and 81% men in the patients, therefore when analyzing these populations, adjustment for age and sex was performed (see Statistical analysis and Results).

### GBA sequencing and classification of GBA variants

*GBA* was fully sequenced as described previously^19^ and the full protocol is available upon request. In brief, we designed molecular inversion probes (MIPs) targeting the coding sequence of *GBA* and performed next-generation sequencing (NGS) post capture. Alignment, variant calling, and annotations were done as previously described^19^ using a standard pipeline. Exons 10 and 11 were also sequenced using Sanger sequencing since the coverage of NGS of these exons was low. Table e-2 (github.com/gan-orlab/GBA_RBD/) details the probes used for the MIPs capture. Classification of *GBA* variants as severe or mild was performed as described previously^5,12^ based on the occurrence of these variants in the severe (type II and type III) and mild (type I) forms of GD. The p.E326K and p.T369M variants, which do not cause GD but have a comparable risk as that of the p.N370S variants in PD,^20,21^ were therefore included in the mild variant group.

### Statistical analysis

To examine the association between *GBA* variants and risk of iRBD and controls, we performed association tests (χ^2^ or Fisher exact test), logistic regression adjusted for sex and age, and burden tests. To examine the association of *GBA* variants with risk of iRBD comparing all controls, we used χ^2^ or Fisher exact tests since there were no available data on age and sex from the controls collected from the literature to perform adjusted logistic regression. We therefore also performed this association using only our in-house European controls, for which data on age and sex were available, using logistic regression model adjusted for age and sex. Of note, having younger controls may result in underestimation of the risk, as some of the young controls with *GBA* variants may develop iRBD or overt neurodegeneration in the future. Therefore, if the statistical adjustment is not complete, the risk estimations that were calculated could be slightly lower (i.e., false-positive results are not likely; rather, underestimated risk is likely). We also performed burden tests using the R package SKAT. Association with AAO and specific types of *GBA* variants (severe or mild) was tested using the nonparametric Kruskal-Wallis test since the group of severe *GBA* variants included only 5 patients. The association with conversion was tested using a χ^2^ test for the total number of conversions, and Kaplan-Meier survival analysis was performed to examine the rate of conversion. All statistical analyses were performed using R or SPSS v24 (IBM, Armonk, NY).

### Standard protocols approvals, registrations, and patient consents

All study participants signed informed consent forms, and the study protocol was approved by the institutional review boards.

### Data availability

Anonymized data will be shared by request from any qualified investigator.

## Results

*GBA* variants are associated with increased risk of iRBD with differential effects of severe and mild variants.

The variants in *GBA* identified in each of the participating centers are detailed in table 1, with a total of 17 distinct variants found in patients and controls (table 2). Table e-1 details the variants found in each of the previously published control populations. Out of 1,061 patients with iRBD, 101 *GBA* variant carriers (9.5%) were identified, compared to 126 out of 3,086 (4.1%) controls (table 2; odds ratio [OR], 2.45; 95% confidence interval [CI], 1.87–3.22; *p* = 1 × 10^−10^). We repeated this analysis using a logistic regression model adjusted for age and sex using the controls with available data (n = 1,317), which yielded very similar results (OR, 2.12; 95% CI, 1.34–3.36; *p* = 0.001). Burden tests using the R package SKAT also yielded similar results (*p* = 2.6 × 10^−6^ using the in-house controls and *p* = 1.7 × 10^−12^ using all controls). Similar to previous observations in PD, different *GBA* variants have different effects on the risk of iRBD. The mild p.N370S variant was found in 20 patients with iRBD (1.9%) compared to 16 (0.5%) controls (OR, 3.69; 95% CI, 1.90–7.14; *p* = 3.5 × 10^−5^), while severe variants (p.L444P, p.D409H, p.W291X, p.H255Q, and p.R131L) were found in 6 (0.6%) patients with iRBD and in 1 (p.L444P, 0.03%) control (OR, 17.55; 95% CI, 2.11–145.9; *p* = 0.0015). Of the 2 polymorphisms known to be risk factors for PD, p.E326K and p.T369M, only p.E326K was associated with iRBD (4.4% vs 1.5% in patients and controls; OR, 3.2; 95% CI, 2.12–4.84; *p* = 6 × 10^−9^), and the carrier frequency of p.T369M was only slightly elevated in iRBD but not statistically significant (1.9% vs 1.7%; OR, 1.13; 95% CI, 0.68–1.89; *p* = 0.6). The carrier frequencies of the p.N370S, p.E326K, and p.T369M variants in gnomAD (gnomad.broadinstitute.org/) in the European population are 0.4%, 2.4%, and 1.9%, respectively, similar to the frequencies in our controls.

**Table 2:**
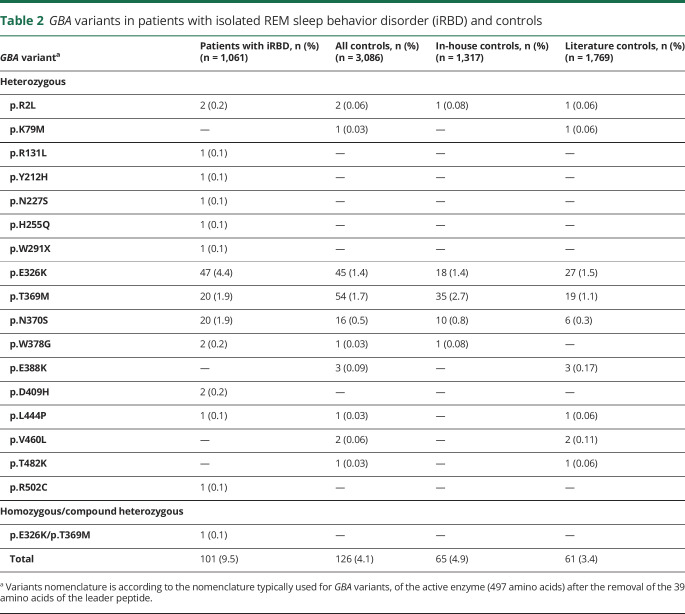
*GBA* variants in patients with isolated REM sleep behavior disorder (iRBD) and controls

### Estimated AAO of iRBD may be affected by the type of *GBA* variant

AAO as reported by the patients could be an unreliable estimate and data were not available for all patients. Therefore, the following results should be considered with caution. Carriers of the severe *GBA* variants had an average AAO of 52.8 ±2.8 years (data were available for 5 out of 6 patients with a severe *GBA* variant), carriers of all other variants had an average AAO of 59.7 ± 9.6 years (data were available for 58 patients), and noncarriers of *GBA* variants had an average AAO of 60.6 ± 9.9 years (data were available for 531 patients). Because there were only 5 patients in the severe variants group, the nonparametric Kruskal-Wallis test was performed, demonstrating a possible association with the type of variant (χ^2^ = 7.083, *df* = 3, *p* = 0.029), which will benefit from replication in a larger sample size.

### Do *GBA* variants affect the rate of conversion of iRBD to overt neurodegenerative diseases?

Data on conversion of iRBD was available for 59 *GBA* variant carriers and 525 noncarriers of *GBA* variants. Of the *GBA* variant carriers, 31 (52.5%) had converted, and in noncarriers 187 (35.6%) had converted (*p* = 0.011). Data on time from iRBD diagnosis to phenoconversion or last follow-up was available for 29 *GBA* variant carriers and for 276 noncarriers. Kaplan-Meier survival analysis suggested that *GBA* variant carriers progressed faster but the difference vs noncarriers of *GBA* variants was not statistically significant (figure). When severe *GBA* variant carriers were compared to mild *GBA* carriers and noncarriers, a possible association was demonstrated, as the Breslow test was statistically significant and the Tarone-Ware test was at near statistical significance, while the log-rank test did not reach statistical significance (figure; Breslow *p* = 0.017, Tarone-Ware *p* = 0.051, log-rank *p* = 0.24).

**Figure:**
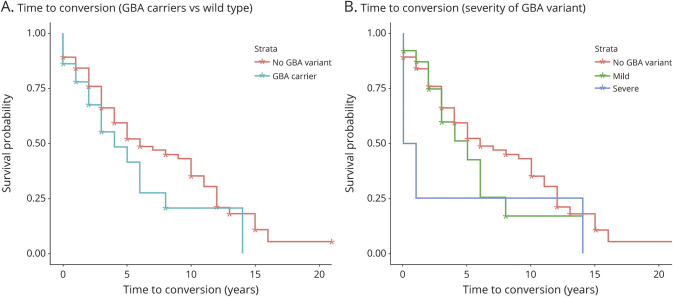
Conversion to overt neurodegenerative disease in patients with iRBD with and without *GBA* variants (A) Survival plot comparing *GBA* variant carriers (blue) and noncarriers (red) from diagnosis until conversion or recent follow-up. Log-rank *p* = 0.13, Breslow *p* = 0.32, Tarone-Ware *p* = 0.22. (B) Survival plot comparing carriers of severe *GBA* variants (blue), mild/other *GBA* variants (green), and noncarriers (red). Log-rank *p* = 0.24, Breslow *p* = 0.017, Tarone-Ware *p* = 0.051.

## Discussion

Our results confirm the association between *GBA* variants and increased risk of iRBD and suggest that severe and mild *GBA* variants have differential effects on risk, similar to previous reports in PD.^5^ These results also suggest that patients with iRBD with severe *GBA* variants may have earlier AAO and may convert faster to overt neurodegenerative disease. However, the results on AAO and conversion should be considered as preliminary only and with caution, due to several limitations discussed below.

Three previous small sample size studies have examined the association between *GBA* variants and iRBD.^15,16,22^ Two of these studies included full sequencing of the gene^15,16^ and the third only examined 2 specific variants (p.N370S and p.L444P).^22^ Due to their size, analyses of specific variants or types of variants, such as severe or mild, were not possible. The current study includes 2 of the previously published cohorts^15,22^ and additional cohorts of European ancestry. With the larger sample size accrued, we were able to demonstrate a much larger risk in carriers of severe *GBA* variants. Given the small numbers of these variants and the wide range of the CIs, the risk estimates may be different in future, larger studies. The current results are in line with previous results from PD, which clearly demonstrated similar relationships between severe and mild *GBA* variants and risk for PD.^5^ Previous studies have also suggested that the type of *GBA* variants may affect PD progression,^13,14^ which is further supported by our preliminary findings on AAO and conversion of iRBD.

In recent years it has been demonstrated that the coding variants p.E326K and p.T369M, which do not cause GD, are risk factors for PD.^20,21,23^ In DLB, the association between p.E326K and risk for the disease is clear, yet it is still unclear whether p.T369M is a risk factor for DLB. Only a few studies that examined p.T369M in DLB have been performed and in most of them there was no association. A multicenter study that included over 700 patients with DLB reported lack of association, and in a GWAS with over 1,700 patients with DLB, only the p.E326K variant was reported to be associated with the disease.^7^ Conversely, recent data from 556 patients with DLB did suggest an association.^24^ The lack of association in the current study in iRBD may also provide further support for lack of association of p.T369M with iRBD and DLB. However, it is important to keep in mind that the association of this variant with PD was only reported in much larger studies^20,21^ due to its lower effect on risk compared to other *GBA* variants. Only much larger studies can determine conclusively whether p.T369M is associated with iRBD and DLB. There was a large difference between the frequency of p.T369M in our in-house controls (2.7%) and the controls from the literature (1.1%), perhaps due to population structure, but the combined frequency (1.7%) is comparable to that seen in the gnomAD European population (1.9%), rendering our results for this variant as likely unbiased.

Our study has several limitations. The possible association between *GBA* variants and rate of conversion reported here, although potentially interesting, should be taken with caution for several reasons. (1) The results include the cohort from Montreal, in which it was previously reported that *GBA* variants are associated with rate of conversion, but it does not include the negative study from Barcelona (data could not be shared). (2) The results are based on a small number of variant carriers (4 patients with a severe *GBA* variant, 25 with other *GBA* variants). Larger studies will be required to determine conclusively whether *GBA* variants are associated with the rate of phenoconversion. An additional potential limitation is that the measured duration from age at diagnosis or iRBD to conversion might not reflect the actual length of disease duration, as patients can remain unaware for many years about their dream-enactment behaviors, especially if they do not have a bed partner or if they do not have very active or violent dreams. The small number of severe *GBA* variants is also a limitation in the risk analysis, as it created a wide CI. Since the effect of severe vs mild variants is in line with previous studies in PD, it is likely that these risk estimates of iRBD are overall correct, yet the precise estimate might change in future, larger studies.

The mechanisms underlying the association between *GBA* variants, the enzyme encoded by *GBA*, glucocerebrosidase (GCase), and the development of neurodegeneration are unknown.^11^ Several mechanisms have been proposed, including interaction of GCase substrates with α-synuclein, which may lead to its accumulation,^25^ changes in the lysosomal membrane composition, which may lead to reduced autophagy and mitophagy,^26,27^ accumulation of misfolded GCase and endoplasmic reticulum stress,^28^ and others. The association with iRBD may suggest that studying these mechanisms in nondopaminergic neuronal models that are involved in RBD could lead to new discoveries and better understanding of these potential mechanisms.

Our results demonstrate that *GBA* variants are associated with increased risk of iRBD. These results may also suggest that severe and mild *GBA* variants may have differential effects on the risk, and possibly on AAO, of iRBD and its conversion to overt neurodegenerative disease. Due to the limitations mentioned above, the latter associations should be considered as preliminary with additional, larger studies on *GBA* in iRBD required to confirm or refute them. One important implication of the association between *GBA* variants and iRBD is the possibility to perform screening for iRBD in healthy *GBA* variant carriers. This may allow for even earlier detection of prodromal neurodegeneration and could be especially useful when home detection of iRBD will be made possible.

## Glossary

AAO: age at onset
CI: confidence interval
DLB: dementia with Lewy bodies
GCase: glucocerebrosidase
GD: Gaucher disease
GWAS: genome-wide association study
iRBD: isolated REM sleep behavior disorder
MIP: molecular inversion probe
MSA: multiple system atrophy
NGS: next-generation sequencing
OR: odds ratio
PD: Parkinson disease
pRBD: probable REM sleep behavior disorder
RBD: REM sleep behavior disorder
